# Metabolic Adaptation of Methanogens in Anaerobic Digesters Upon Trace Element Limitation

**DOI:** 10.3389/fmicb.2018.00405

**Published:** 2018-03-13

**Authors:** Babett Wintsche, Nico Jehmlich, Denny Popp, Hauke Harms, Sabine Kleinsteuber

**Affiliations:** ^1^Department of Environmental Microbiology, Helmholtz Centre for Environmental Research—Helmholtz-Zentrum für Umweltforschung (UFZ), Leipzig, Germany; ^2^Department of Molecular Systems Biology, Helmholtz Centre for Environmental Research—Helmholtz-Zentrum für Umweltforschung (UFZ), Leipzig, Germany

**Keywords:** methanogenic pathways, metaproteome, trace metals, biogas process, *mcrA*, *Methanosarcina*, *Methanoculleus*

## Abstract

Anaerobic digestion (AD) is a complex multi-stage process relying on the activity of highly diverse microbial communities including hydrolytic, acidogenic and syntrophic acetogenic bacteria as well as methanogenic archaea. The lower diversity of methanogenic archaea compared to the bacterial groups involved in AD and the corresponding lack of functional redundancy cause a stronger susceptibility of methanogenesis to unfavorable process conditions such as trace element (TE) deprivation, thus controlling the stability of the overall process. Here, we investigated the effects of a slowly increasing TE deficit on the methanogenic community function in a semi-continuous biogas process. The aim of the study was to understand how methanogens in digester communities cope with TE limitation and sustain their growth and metabolic activity. Two lab-scale biogas reactors fed with distillers grains and supplemented with TEs were operated in parallel for 76 weeks before one of the reactors was subjected to TE deprivation, leading to a decline of cobalt and molybdenum concentrations from 0.9 to 0.2 mg/L, nickel concentrations from 2.9 to 0.8 mg/L, manganese concentrations from 38 to 18 mg/L, and tungsten concentrations from 1.4 to 0.2 mg/L. Amplicon sequencing of *mcrA* genes revealed *Methanosarcina* (72%) and *Methanoculleus* (23%) as the predominant methanogens in the undisturbed reactors. With increasing TE limitation, the relative abundance of *Methanosarcina* dropped to 67% and a slight decrease of acetoclastic methanogenic activity was observed in batch tests with ^13^C-methyl-labeled acetate, suggesting a shift toward syntrophic acetate oxidation coupled to hydrogenotrophic methanogenesis. Metaproteome analysis revealed abundance shifts of the enzymes involved in methanogenic pathways. Proteins involved in methylotrophic and acetoclastic methanogenesis decreased in abundance while formylmethanofuran dehydrogenase from *Methanosarcinaceae* increased, confirming our hypothesis of a shift from acetoclastic to hydrogenotrophic methanogenesis by *Methanosarcina*. Both *Methanosarcina* and *Methanoculleus* increased the abundance of N5-methyltetrahydromethanopterin-coenzyme M methyltransferase and methyl-coenzyme M reductase. However, these efforts to preserve the ion motive force for energy conservation were seemingly more successful in *Methanoculleus*. We conclude that both methanogenic genera use different strategies to stabilize their energy balance under TE limitation. *Methanosarcina* switched from TE expensive pathways (methylotrophic and acetoclastic methanogenesis) to hydrogenotrophic methanogenesis. *Methanoculleus* showed a higher robustness and was favored over the more fastidious *Methanosarcina*, thus stabilizing reactor performance under TE limitation.

## Introduction

Anaerobic digestion (AD) is a widespread and effective way for recycling organic waste and biomass residues while producing biogas as renewable energy carrier. Biogas production is a scalable, technically simple and low-cost technology and has therefore a huge potential for renewable energy supply in developing countries (Surendra et al., 2014). In a renewable energy system, it can contribute to all energy sectors (electricity, heating, and mobility) and complement fluctuating renewable energy sources such as wind and solar power (Patterson et al., 2011; Raboni et al., 2015).

AD is a complex multi-stage process relying on the activity of highly diverse microbial communities (Weiland, 2010). The process can be divided in four main phases: hydrolysis, acidogenesis, acetogenesis and methanogenesis. Methanogenesis is exclusively performed by distinct groups of archaea. Seven phylogenetic orders of methanogens (all belonging to the phylum *Euryarchaeota*) have been described so far (*Methanobacteriales, Methanococcales, Methanomassiliicoccales, Methanomicrobiales, Methanosarcinales, Methanocellales*, and *Methanopyrales*) and all but *Methanopyrales* are ascertainable in biogas processes (Borrel et al., 2013).

Methane can be produced via three different pathways. Hydrogenotrophic methanogens produce methane from carbon dioxide and hydrogen or formate. This pathway is performed by the cultivated methanogens of the orders *Methanobacteriales, Methanococcales, Methanomicrobiales, Methanocellales*, and *Methanopyrales* as well as some members of the *Methanosarcinales*. Methylotrophic methanogens can grow on methylated compounds like methanol or methylamines by dismutation (Whitman et al., 2006). Acetate is directly dismutated to methane and carbon dioxide by acetoclastic methanogens. Cultivated acetoclastic and methylotrophic methanogens are all members of the order *Methanosarcinales*. The recently described genus *Methanomassiliicoccus* belonging to the order *Methanomassiliicoccales* (class *Thermoplasmata*) is an exception (Borrel et al., 2013). It is capable of reducing methanol with hydrogen (Dridi et al., 2012) and might also use methylamines as methanogenic substrate (Poulsen et al., 2013). The reduction of methanol to methane with hydrogen was also described for *Methanosphaera stadtmanae*, which belongs to the *Methanobacteriales* (Miller and Wolin, 1985). Recent findings from metagenome analyses suggest that the actual metabolic and phylogenetic diversity of methanogens might be much higher and comprise a new class of *Euryarchaeota* (“*Methanofastidiosa*”—Nobu et al., 2016) or even other archaeal phyla (“*Bathyarchaeota*”—Evans et al., 2015; “*Verstraetearchaeota*”—Vanwonterghem et al., 2016).

Compared to the bacterial groups involved in AD, the lower diversity and the lack of functional redundancy among methanogenic archaea causes the susceptibility of methanogenesis to unfavorable process conditions such as trace element (TE) deprivation, thus determining the stability of the whole process (Demirel, 2014). The need for TE and the effects of TE limitation on methanogens and reactor performance have been addressed by various studies and reviews (Park et al., 2010; Demirel and Scherer, 2011; Choong et al., 2016). Cobalt, molybdenum, nickel, selenium and tungsten next to iron are known as essential TE for methanogens as shown by studies on their elemental composition (Scherer et al., 1983), their metallo-enzymes (Glass and Orphan, 2012; Choong et al., 2016) and the effect of stimulation by TE (Takashima et al., 1990). For instance, nickel is one of the most important TE for methanogens (Diekert et al., 1981) and was shown to enhance acetate utilization rates (Speece et al., 1983) and increase methane yields in maize silage-fed batch reactors by about 27% (Evranos and Demirel, 2015). Changes of AD reactor performance due to changing TE supplementation are mainly explained on the basis of the methanogenesis step (Park et al., 2010; Demirel and Scherer, 2011; Ariunbaatar et al., 2016; Choong et al., 2016).

Further studies are required to understand how methanogens react to TE deprivation specifically by adapting their metabolism and energy balance especially under limiting conditions. Here, we investigated the effects of a slowly increasing TE deficit on the methanogenic community function in a semi-continuous AD process. After parallel operation of two lab-scale reactors that were well supplied with TE, the TE supplementation of one reactor was stopped, resulting in a decline of TE concentrations to insufficient levels. As shown in our previous study (Wintsche et al., 2016), the slowly decreasing TE supply did not affect reactor efficiency, although shifts of the methanogenic community composition and presumably shifts in the methanogenic pathways were indicated by community fingerprinting of metabolic marker genes and their transcripts. The aim of the present study was to use metaproteomics and metabolite analyses with ^13^C-labeled tracers to understand in more detail how methanogens cope with TE limitation and sustain their growth and metabolic activity leading to AD reactor stability.

## Materials and methods

### Laboratory-scale biogas reactors and sampling

Two identical continuous stirred tank reactors (working volume: 10 L) designated R1 and R2 were operated under mesophilic conditions for 93 weeks as described by Wintsche et al. (2016). The feedstock was dried distillers grains with solubles and the reactors were supplemented with a commercial iron additive and a TE mixture containing cobalt, nickel, molybdenum and tungsten as described by Schmidt et al. (2013). The reactors were operated at an organic loading rate of 5 g_VS_ L^−1^ d^−1^ (VS – volatile solids) resulting in a hydraulic retention time of 25 d. Both reactors were operated in parallel for 76 weeks before starting the experimental period in which the TE supply to R2 was altered by omitting the TE solution and reducing the supply of the iron additive from 2.57 to 0.86 g per day. This altered feeding scheme led to a decline of cobalt and molybdenum concentrations from around 0.9 to 0.2 mg/L, nickel concentrations from 2.9 to 0.8 mg/L, manganese concentrations from 38 to 18 mg/L, and tungsten concentrations from 1.4 to 0.2 mg/L from week 65 to 84. For a detailed description of the reactor setup, operational conditions and detailed measurements and modeling of TE depletion, see Wintsche et al. (2016).

Samples for batch experiments with ^13^C-labeled acetate and proteome analysis were taken at four sampling times (week 65, 77, 80, and 84). Samples for DNA extraction were taken in week 74, 77, 80 and 84. The first sample was taken before the TE supplementation was stopped to ensure comparability for both undisturbed reactors. The next samples were taken one, 4 and 8 weeks after omitting the TE supply of R2.

### Methanogenic community analysis

The methanogenic communities of both reactors at the four sampling times were analyzed by amplicon sequencing of *mcr*A genes. Reactor samples were stored at −20°C until DNA extraction. DNA was extracted with PowerSoil DNA Isolation Kit (MoBio Laboratories Inc., USA) according to the manufacturers' instructions. PCR amplification of *mcrA* genes was performed as described previously (Steinberg and Regan, 2008). Amplicons were sequenced using the 454 pyrosequencing platform GS Junior (Roche) according to Ziganshin et al. (2013). Raw sequences were analyzed with QIIME 1.9.1 Virtual Box release (Caporaso et al., 2010) as described by Popp et al. (2017). Briefly, sequences were quality filtered and chimeric sequences were removed. Sequences were clustered into operational taxonomic units based on 97% sequence identity and were taxonomically classified against a custom database compiled of *mcrA* sequences deposited in the Functional Gene Repository (Fish et al., 2013) using the RDP Classifier 2.2 (Wang et al., 2007). De-multiplexed raw sequences were deposited under the EMBL-EBI study accession number PRJEB21972 (http://www.ebi.ac.uk/ena/data/view/PRJEB21972).

### Batch experiments with ^13^C-labeled acetate

Labeling experiments at four sampling times (I – week 65, II – week 77, III – week 80, IV – week 84) were done by transferring 1.7 L sludge from each reactor into 2-L Duran bottles purged with nitrogen. The bottles were closed, the headspace purged with biogas (61% CH_4_, 39% CO_2_, 50 ppm H_2_S, 50 ppm H_2_, 50 ppm O_2_) and connected to a gas sampling bag. The bottles were incubated for 3 days at 37°C without feeding to reduce the high organic carbon pool within the samples. Bottles were swiveled daily.

^13^C-labeled acetate (0.5 M) was applied as sodium salt. Carboxyl-labeled acetate (Sigma-Aldrich, isotopic purity 99 atom % ^13^C) and methyl-labeled acetate (Sigma-Aldrich, isotopic purity 99 atom % ^13^C) were fed in separate batch cultures. All solutions were prepared with sterile anoxic distilled water in glass vials. The closed vials were purged with nitrogen. Five 50-mL serum bottles for each labeled substrate and each reactor sample were prepared. All bottles were filled with 25 mL reactor sludge and closed airtight in an anaerobic chamber (97% N_2_ and 3% H_2_ atmosphere); then the headspaces were purged with biogas (composition as described above) outside the anaerobic chamber. The batch cultures were fed with 500 μL of ^13^C-acetate solution via a syringe. Immediately after feeding and then every 2 h, one bottle per substrate was processed for gas and proteome analyses. The produced gas was released via a cannula and the volume measured by a U-tube manometer as described by Porsch et al. (2015). Gas composition was determined in triplicates by gas chromatography according to Sträuber et al. (2015). Analyses and calculation of labeled gas ratios (^13^C-CO_2_ to ^12^C-CO_2_ and ^13^C-CH_4_ to ^12^C-CH_4_) were done by gas chromatography mass spectrometry (MS) according to Popp et al. (2016). For proteome analysis, 500 μL of the sludge were centrifuged at maximum speed and the supernatant was discarded. The pellet was stored at −20°C until protein extraction.

### Protein extraction and preparation

The methanogenic communities of both reactors at the four sampling weeks were analyzed using metaproteomics. For reactor R2, 10 batch cultures per sampling week were sampled for protein extraction. For the control reactor R1, 10 batch cultures in week 65 and three batch cultures each in week 77, 80, and 84 were analyzed for their metaproteome (see Supplementary Material, Data Sheet 3). To each sample pellet, 5 mL sodium dodecyl sulfate (SDS) buffer (1.25% w/v SDS, 0.1 M Tris/HCl pH 6.8, 20 mM dithiotreitol) was added and incubated for 1 h at room temperature. Afterwards, samples were centrifuged (30 min at 10,000 × g and 4°C) and the supernatant was collected and filtered through a nylon mesh with a pore size of 0.45 mm. The filtrate was mixed with the equal volume of phenol solution (10 g/mL) and incubated at room temperature for 15 min. Samples were centrifuged and the phenol phase was collected. The water phase was again mixed with the equal volume of phenol solution, incubated 15 min at room temperature with shaking and then centrifuged (12 min at 10,000 × g and 4°C). Both phenol phases were pooled and washed twice with the equal volume of Millipore water for 15 min. After centrifugation (12 min at 10,000 × g and 4°C), the water phase was discarded and the proteins in the phenol phase were precipitated over night at −20°C with ice-cold ammonium acetate (100 mM ammonium acetate in methanol, five-fold, stored at −20°C). Protein pellets were obtained by centrifugation (12 min at 10,000 × g and 4°C). Protein pellets were resuspended in 20 μL SDS sample buffer (2% w/v SDS, 2 mM β-mercaptoethanol, 4% v/v glycerol, 40 mM Tris/HCl pH 6.8, 0.01% w/v bromophenol blue), heated at 90°C for 4 min and separated for 10 min by electrophoresis in a 12% SDS polyacrylamide gel (4% stacking gel, 12% separating gel). After electrophoresis, the gels were stained with colloidal Coomassie brilliant blue (Merck). The gel area containing the protein mixture of each sample was cut out in one piece, destained, dehydrated and proteolytically cleaved overnight at 37°C by trypsin (Promega). Extracted peptides were desalted using C18 ZipTip columns (Merck Millipore). Peptide lysates were dissolved in 0.1% formic acid and analyzed by liquid chromatography MS.

### Mass spectrometry-based proteome analyses

The peptide lysates were separated on a UHPLC system (Ultimate 3000, Dionex/Thermo Fisher Scientific, Idstein, Germany). Five microliter samples were first loaded for 5 min on the pre-column (μ-pre-column, Acclaim PepMap, 75 μm inner diameter, 2 cm, C18, Thermo Scientific) at 4% mobile phase B (80% acetonitrile in Nanopure water with 0.08% formic acid), 96% mobile phase A (Nanopure water with 0.1% formic acid), then eluted from the analytical column (PepMap Acclaim C18 LC Column, 25 cm, 3 μm particle size, Thermo Scientific) over a 150 min non-linear gradient of mobile phase B (4–55% B).

MS was performed on an Orbitrap Fusion MS (Thermo Fisher Scientific, Waltham, MA, USA) with a TriVersa NanoMate (Advion, Ltd., Harlow, UK) source in LC chip coupling mode. The MS was set at cycle time of 3 s used for MS/MS scans with higher energy collision dissociation (HCD) at normalized collision energy of 28%. MS scans were measured at a resolution of 120,000 in the scan range of 350–2,000 m/z. MS ion count target was set to 4 × 10^5^ at an injection time of 100 ms. Ions for MS/MS scans were isolated in the quadrupole with an isolation window of 1.6 Da and were measured with a resolution of 15,000 in the scan range of 350–1,400 m/z. The dynamic exclusion duration was set to 30 s with a 10 ppm tolerance. Automatic gain control target was set to 6 × 10^4^ with an injection time of 150 ms using the underfill ratio of 1%.

### Bioinformatics analysis

Protein identification was performed using the Proteome Discoverer (v1.4.0.288, Thermo Scientific). The acquired MS/MS spectra (^*^.raw files) were searched using the Sequest HT algorithm against the database provided by Kohrs et al. (2015) extended with Uniprot entries for methanogens and several syntrophic bacteria. Search parameters were set as follows: tryptic cleavage, maximum of two missed cleavage sites, a precursor mass tolerance threshold of 10 ppm and a fragment mass tolerance threshold of 0.02 Da. In addition, carbamidomethylation at cysteine was selected as a static and oxidation of methionine as a variable modification. Only peptides that passed the false discovery rate (FDR) of <1% and peptide rank = 1 were considered for protein identification. Label-free quantification was done using peptide spectral matching (PSM). The PROteomics results Pruning & Homology group ANotation Engine (PROPHANE) was used to calculate protein abundances based on the normalized spectral abundance factor (NSAF; von Bergen et al., 2013) and to assign proteins to their taxonomic and functional groups (www.prophane.de). Taxonomic assignment was done by BLASTp v2.2.28+ (*E*-value: ≤0.001). Functional classification was based on TIGRFAM, Pfam-A and cluster of orthologous groups (COG) (*E*-value: ≤0.01).

Data analysis was focused on methanogenesis enzymes of the families *Methanomicrobiaceae* and *Methanosarcinaceae*; any bacterial or other archaeal hits were excluded from further analyses. Transformation, normalization and statistical analysis of protein group intensity data were performed using R (v 2.15.02) and “ggplot2” (v 0.9.3.1) (Wickham, 2009).

## Results

### Community composition and dynamics of methanogens

**Figure 1:**
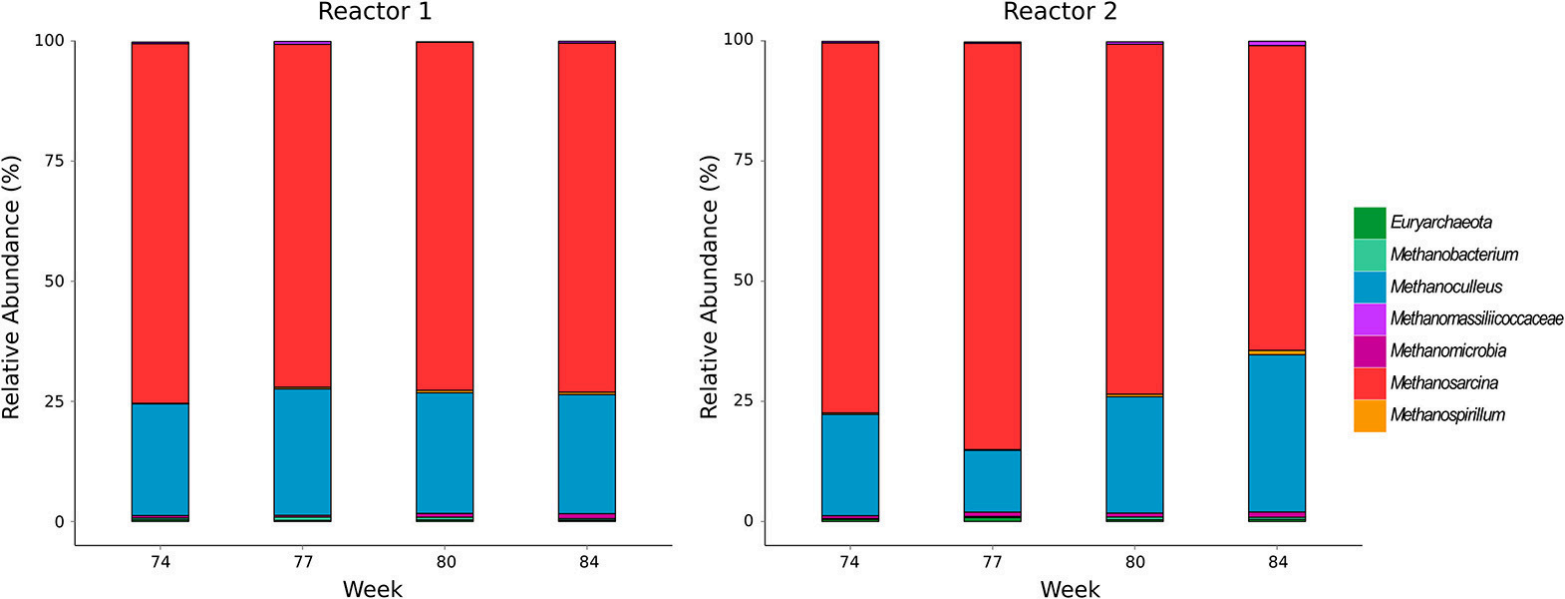
Community composition of methanogens determined by amplicon sequencing of *mcr*A genes.

The community composition of methanogens was examined by amplicon sequencing of the *mcr*A gene. Number of sequence reads, operational taxonomic units and rarefaction curves are shown in the Supplementary Material (Data Sheet 2, Figure S1). *Methanosarcina* spp. and *Methanoculleus* spp. dominated in both reactors while negligible abundances of *Methanospirillum, Methanobacterium* and other methanogens not classified to the genus level were detected (Figure 1). The methanogenic community in the undisturbed reactor R1 underwent minor fluctuations over all sampling times (*Methanosarcina* 71–75%; *Methanoculleus* 23-26%). Reactor R2 showed a comparable community composition in week 74 during sufficient TE supply (*Methanosarcina* 77%; *Methanoculleus* 21%), while the relative abundance of *Methanoculleus* dropped to 13% after initiating TE deprivation in week 77, then recovered until week 80 to 24% and increased further to 33% in week 84. The relative abundance of *Methanosarcina* in R2 behaved inversely, suggesting an adaptation of the methanogenic community to the incremental TE depletion.

### Active methanogenic pathways

Methanogenic pathways were analyzed by feeding ^13^C-methyl-labeled acetate to batch cultures set up using reactor content, and recording the formation of ^13^C-labeled methane. The amount of ^13^C-labeled methane formed from the ^13^C-methyl-labeled acetate fed to each batch culture (0.25 mmol) was calculated based on the measured methane volume and the ratio of ^13^C-CH_4_ to ^12^C-CH_4_ as determined by GC-MS. In the first two sampling times (weeks 65 and 77) the batch cultures set up from both reactors produced similar amounts of ^13^C-labeled methane within 8 h after feeding methyl-labeled acetate. In sampling week 80 samples from reactor R2 produced slightly more labeled methane than those from reactor R1, whereas in week 84 a slight drop of the labeled methane was observed in reactor R2 compared to reactor R1 (Figure S2). This data indicates a partial metabolic shift of the active methanogenic pathways from acetoclastic methanogenesis toward syntrophic acetate oxidation (SAO) coupled to hydrogenotrophic methanogenesis.

**Figure 2:**
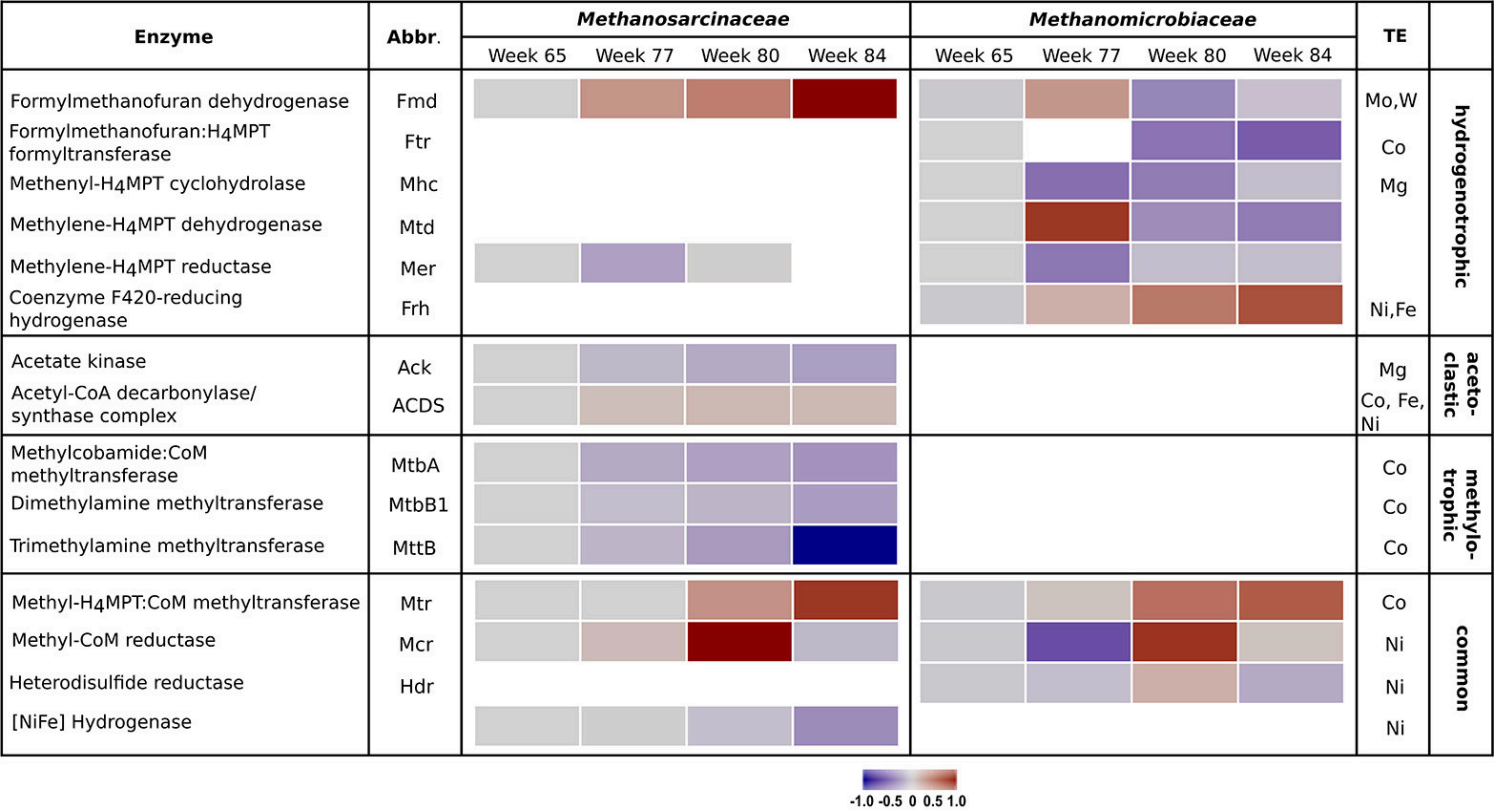
Heatmap of enzyme abundances of the methanogenic pathways employed by *Methanosarcinaceae* and *Methanomicrobiaceae* over the four sampling times in reactor R2. For each enzyme, the specific methanogenic pathway and the required trace elements are given. Gray bars indicate initial conditions, blue bars declining protein abundances, and red bars increasing protein abundances. Missing bars indicate enzymes that were not detected.

The metabolic shift was analyzed in detail by examining the enzyme abundances of the different methanogenic pathways. Results of the proteome analysis are provided in the Supplementary Material (Data Sheet 3). Table 1 lists all detected enzymes, their reactions and enzyme classification. Similar to the community composition as detected by *mcrA* amplicon sequencing, the enzyme abundances in the control reactor R1 underwent minor fluctuations over the four sampling times (Figure S3). In contrast, reactor R2 showed remarkable trends linked to TE deprivation as illustrated in Figure 2. In the beginning (week 77) the declining TE concentrations caused lower abundances of several enzymes involved in hydrogenotrophic methanogenesis of the *Methanomicrobiaceae*, such as methenyl-H_4_MPT cyclohydrolase (Mhc), methylene-H_4_MPT reductase (Mer) and methyl-CoM reductase (Mcr). However, Mcr abundance increased again in week 80. Other enzymes of the *Methanomicrobiaceae* became more abundant in week 77 and decreased later in abundance, such as formylmethanofuran dehydrogenase (Fmd) and methylene-H_4_MPT dehydrogenase (Mtd). Abundance of the cobalt-dependent formylmethanofuran:H_4_MPT formyltransferase (Ftr) decreased as well. Only two enzymes of *Methanomicrobiaceae* were more abundant at lower TE concentrations over all sampling times: coenzyme F_420_-reducing hydrogenase (Frh) and methyl-H_4_MPT:CoM methyltransferase (Mtr). For the *Methanosarcinaceae*, abundance shifts were more pronounced. Abundances of cobalt-dependent enzymes involved in methylotrophic methanogenesis declined as well as acetate kinase (Ack) (involved in acetoclastic methanogenesis) and [NiFe] hydrogenase. Surprisingly, the nickel-dependent acetyl-CoA decarboxylase/synthase complex (ACDS) slightly increased in its abundance with declining TE concentrations. Only formylmethanofuran dehydrogenase (Fmd) and methyl-H_4_MPT:CoM methyltransferase (Mtr) showed increasing abundances, the latter as observed for the *Methanomicrobiaceae*.

**Table 1:** Reactions of the hydrogenotrophic, acetoclastic and methylotrophic methanogenesis and all involved enzymes detected in the proteome analysis.

**Reaction**	**Enzyme**	**Abbr**.	**Equation**	**Enzyme class**	
1	Formylmethanofuran dehydrogenase	Fmd	*CO*_2_ + *methanofuran* + *reduced acceptor ⇌formylmethanofuran* + *H*_2_*O* + *oxidized acceptor*	1.2.99.5	Hydrogenotrophic
2	Formylmethanofuran:H_4_SPT formyltransferase	Ftr	$\begin{array}{l} formylmethanofuran+H_{4}MPT+H^{+}\\ \;⇌5-formyl-H_{4}MPT\\ \;+methanofuran \end{array}$	2.3.1.101	
3	Methenyl-H_4_SPT cyclohydrolase	Mch	$\begin{array}{l} 5-formyl-H_{4}MPT+H^{+}\\ \;⇌5,10-methenyl-H_{4}MPT+H_{2}O \end{array}$	3.5.4.27	
4	F_420_-dependent methylene-H_4_SPT dehydrogenase	Mtd	a) $\begin{array}{l} 5,10-methenyl-H_{4}MPT+red.\;F_{420}+H^{+}+\;⇌\\ \;5,10-methylene-H_{4}MPT+ox.\;F_{420} \end{array}$ b) $\begin{array}{l} 5,10-methenyl-H_{4}MPT+H_{2}+\;⇌5,10\\ -methylene-H_{4}MPT+H^{+} \end{array}$	1.5.98.1/ 1.12.98.2	
5	F_420_-dependent methylene-H_4_SPT reductase	Mer	5, 10 − *methylene* − *H*_4_*MPT* + *reduced coenzyme F*_420_ ⇌ 5 − *methyl* − *H*_4_*MPT* + *coenzyme F*_420_	1.5.98.2	
6	Methyl-H_4_MPT:coenzyme M methyltransferase	Mtr	a) 5 − *methyl* − *H*_4_*MPT* + *coenzyme M ⇌ methyl* − *coenzyme M* + *H*_4_*MPT* b) 5 − *methyl* − *H*_4_*MPT* + *cob*(*I*)*alamine ⇌ methyl* − *cob*(*III*)*alamine* + *H*_4_*MPT* c) *methyl* − *cob*(*III*)*alamine* + *coenzyme M ⇌ methyl* − *coenzyme M* + *cob*(*I*)*alamine*	2.1.1.86	
7	Factor F_420_-reducing hydrogenase	Frh	*F*_420_ (*oxidized*) + *H*_2_ ⇌ *F*_420_*H*_2_	1.12.98.1	
8	Acetate kinase	Ack	*ATP* + *acetate ⇌ ADP* + *acetyl phosphate*	2.7.2.1	Acetoclastic
9	Acetyl-CoA decarbonylase/synthase complex	ACDS	$\begin{array}{l} acetyl-CoA+H_{4}SPT+H_{2}O\\ \;⇌5-methyl-H_{4}SPT+CO_{2}+H^{+} \end{array}$	2.1.1.-	
10	Trimethylamine methyltransferase	MttB	*trimethylamine* + *MttC* + *H*^+^ ⇌ *methyl* − *MttC* + *dimethylamine*	2.1.1.250	Methylotrophic
11	Dimethylamine methyltransferase	MtbB1	*dimethylamine* + *MtbC* + *H*^+^ ⇌ *methyl* − *MtbC* + *monomethylamine*	2.1.1.249	
12	Methylamine methyltransferase	MtbA	*methyl* − *Mt*(*t, b, m*)*C* + *HS* − *CoM* ⇌ *methyl* − *S* − *CoM* + *Mt*(*t, b, m*)*C*	2.1.1.247	
13	Methyl-CoM reductase	Mcr	*methyl* − *CoM* + *CoB ⇌ CoM* − *S* − *S* − *CoB* + *CH*_4_	2.8.4.1	x
14	Heterodisulfide reductase	Hdr	*CoM* − *S* − *S* − *CoB ⇌ CoM* − *SH* + *CoB* − *SH*	1.8.98.1	

*Enzymes, their abbreviations, reaction equations and the corresponding enzyme class are given. x, common to all methanogenic pathways; H_4_MPT, tetrahydromethanopterin; H_4_SPT, tetrahydrosarcinapterin*.

**Figure 3:**
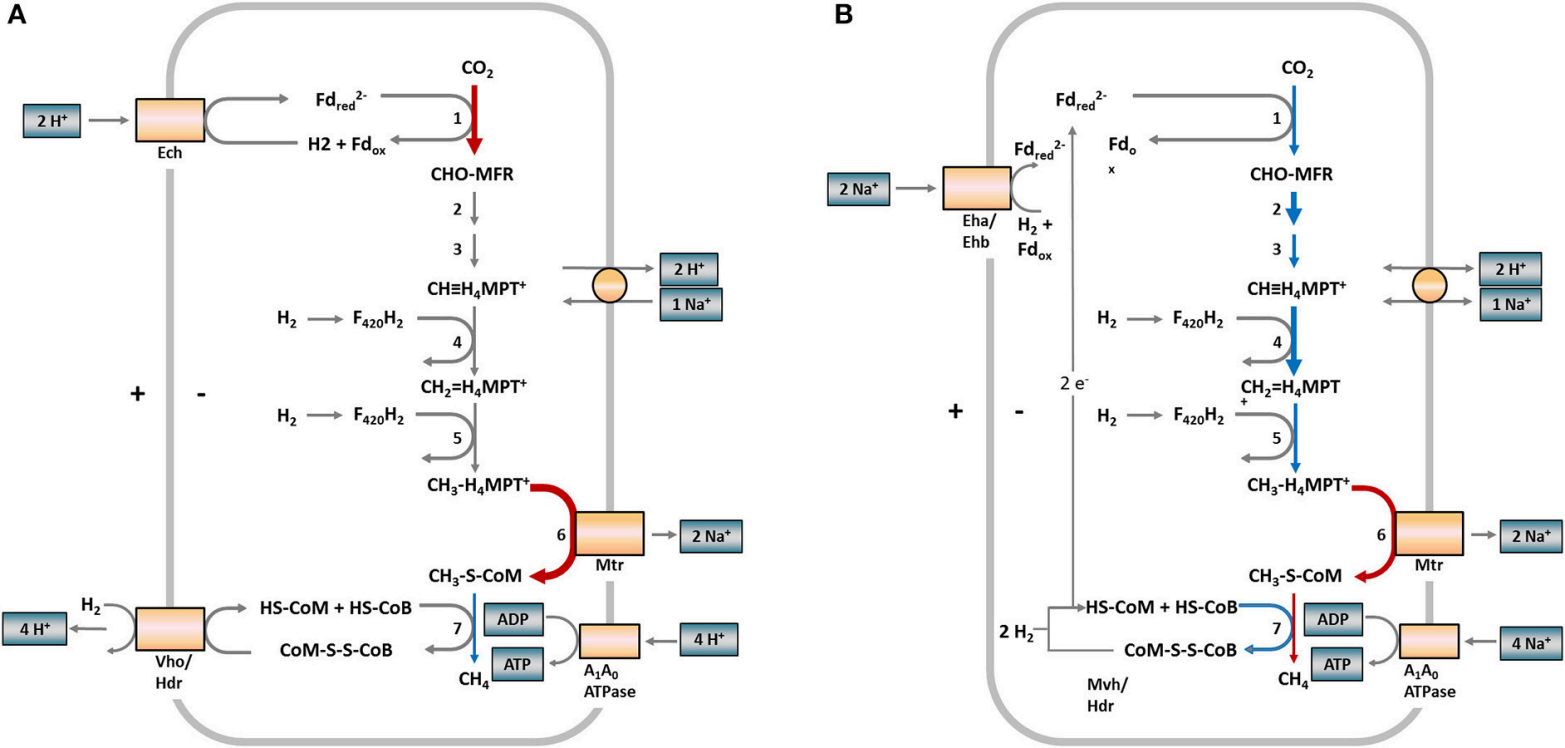
Scheme of hydrogenotrophic methanogenesis in *Methanosarcinaceae* **(A)** and *Methanomicrobiaceae* **(B)** and observed protein abundance shifts in samples from reactor R2 (week 84). The numbers at the reaction arrows correspond to the reaction numbers in Table 1. Colored arrows indicate changing protein abundances between week 65 and 84. Red arrows indicate increasing protein abundances, blue arrows decreasing protein abundances, gray arrows show proteins not detected. Arrow thickness indicates the protein abundances. Thick = more abundant, thin = low abundant. **(A)** In *Methanosarcina*, the first and last steps of methanogenesis are chemiosmotically coupled and ATP generation is driven by a proton motive force. **(B)** In *Methanoculleus*, the first and last steps of methanogenesis are coupled by flavin-based electron bifurcation and ATP generation is driven by a sodium motive force. Fd, ferredoxin; MFR, methanofuran; H_4_MPT, tetrahydromethanopterin; HS-CoM, coenzyme M; HS-CoB, coenzyme B; Ech, FeS hydrogenase; Vho/Hdr, F_420_ non-reducing hydrogenase/heterodisulfide reductase; Eha/Ehb, energy-converting hydrogenase complex; Mvh/Hdr, methyl-viologen-reducing hydrogenase (modified according to Thauer et al., 2008).

For a better overview, Figure 3 depicts the hydrogenotrophic pathways of both methanogens in a metabolic scheme highlighting the abundance shifts of involved enzymes at the last sampling time (week 84) for R2. A corresponding scheme for R1 is presented in Figure S4 (Supplementary Material).

## Discussion

Our previous study on the effects of TE deprivation in AD suggested that limiting concentrations of Co, Mn, Mo, Ni and W cause activity shifts within the methanogenic communities as detected by T-RFLP profiling of *mcrA* transcripts (Wintsche et al., 2016). The major genera affected in the lab-scale biogas reactors investigated were *Methanosarcina* and *Methanoculleus*. The present study confirms these two genera as dominant methanogens by amplicon sequencing of *mcrA* genes, which is a more precise method than T-RFLP analysis. The TE decline in reactor R2 caused only minor shifts in the methanogenic community composition. This observation confirms our previous results that the effects of TE deprivation were more pronounced on the RNA level than on the DNA level (Wintsche et al., 2016).

*Methanosarcina* is a versatile, multipotent methanogen able to degrade diverse substrates via acetoclastic, methylotrophic or hydrogenotrophic methanogenesis (Conklin et al., 2006; De Vrieze et al., 2012). *Methanoculleus* is a hydrogenotrophic methanogen that can also act as syntrophic partner of syntrophic acetate oxidizing bacteria (SAOB). Based on the T-RFLP patterns of *mcrA* transcripts, we hypothesized in Wintsche et al. (2016) a relative activity increase of *Methanoculleus* over *Methanosarcina* and a shift from acetoclastic to hydrogenotrophic methanogenesis in *Methanosarcina* as a consequence of TE deprivation. This hypothesis is supported by the results of the tracer experiment with ^13^C-labeled acetate in the present study. The central AD intermediate acetate is degraded by acetoclastic methanogens or by SAOB. Degradation of ^13^C-methyl-labeled acetate via acetoclastic methanogenesis maintains the label in the methyl moiety, leading to the formation of ^13^C-methane, while SAOB convert acetate completely to CO_2_. Consequently, a shift from acetoclastic methanogenesis to SAO would be reflected in a decline of the methane labeling ratio. The conditions in our control reactor R1 and the undisturbed reactor R2 during full TE supplementation (low concentrations of H_2_S, VFA and low total ammonium nitrogen) were characteristic of AD processes dominated by acetoclastic methanogenesis (Karakashev et al., 2005, 2006; Wintsche et al., 2016). With ongoing TE depletion in reactor R2, the amount of ^13^CH_4_ decreased, indicating a partial shift from acetoclastic methanogenesis to SAO coupled to hydrogenotrophic methanogenesis. In accordance with this observation, the arising reactor conditions in R2 (increasing concentrations of H_2_S, H_2_, VFA concentrations and total ammonium nitrogen – Wintsche et al., 2016) are known to favor hydrogenotrophic over acetoclastic methanogenesis (Schnürer et al., 1994; Karakashev et al., 2005, 2006).

To analyze in more detail at the metabolic level how the major methanogens *Methanosarcina* and *Methanoculleus* cope with TE deprivation, protein abundances of the enzymes involved in methanogenic pathways were examined via proteome analysis of the dominant families *Methanosarcinaceae* and *Methanomicrobiaceae*. Abundances of proteins involved in methylotrophic methanogenesis by *Methanosarcinaceae* decreased. The degradation of methylated compounds proceeds via a very specific pathway including individual methyltransferases and corrinoid-binding proteins. These are specific for their respective substrates and exhibit little or no activity with other methylotrophic substrates (van der Meijden et al., 1983; Burke and Krzycki, 1997; Ferguson and Krzycki, 1997; Wassenaar et al., 1998). The respective enzymes MttB, MtbB1, and MtbA (Table 1) depend on cobalt (corrinoid-containing) and seem to be affected heavily by decreasing cobalt concentrations.

Two enzymes involved in acetoclastic methanogenesis were detected for *Methanosarcinaceae* – the Ack required for acetate activation and the acetyl-CoA decarbonylase/synthase complex (ACDS) that cleaves the C-C and C-S bonds in the acetyl moiety of acetyl-CoA, oxidizes the carbonyl group to CO_2_ and transfers the methyl group to tetrahydrosarcinapterin. The decreasing Ack abundance in R2 could be a hint for decreasing activity of acetoclastic methanogenesis. In contrast, ACDS abundance was relatively stable, which could be explained by the carbon assimilation function of this enzyme complex that is also required during autotrophic growth under hydrogenotrophic conditions (Gencic et al., 2010). *Methanosarcinaceae* might upregulate the synthesis of ACDS subunits to ensure assimilation pathways upon switching from acetoclastic to hydrogenotrophic methanogenesis.

The abundance of formylmethanofuran dehydrogenase (Fmd), which is involved in the first step of hydrogenotrophic methanogenesis and requires Mo or W (Vorholt and Thauer, 2002), strongly increased in *Methanosarcinaceae*, confirming our hypothesis of a pathway switch in methanogenesis. Furthermore, the cobalt-dependent Mtr and Mcr increased in their abundances during TE deprivation. Mtr is a membrane-associated, corrinoid-containing enzyme and drives an energy-conserving ion pump (Gottschalk and Thauer, 2001). Mcr requires the prosthetic group F_430_, which contains Ni as central atom (Whitman and Wolfe, 1980). In week 84, Mtr stayed at an increased level whereas Mcr decreased strongly below the level of week 65 (Figure 2). Further, *Methanosarcinaceae* suffered nickel limitation as visible by the decreasing abundance of [NiFe] hydrogenase without the ability to use Ni-free hydrogenases (Thauer et al., 2010).

*Methanomicrobiaceae* can only perform hydrogenotrophic methanogenesis and are also affected by TE deprivation. Several enzymes of the *Methanomicrobiaceae* increased in abundance and stayed more abundant in week 84 compared to the *Methanosarcinaceae*. This indicates an advantage of *Methanomicrobiaceae* over *Methanosarcinacaea*.

*Methanosarcinaceae* as well as *Methanomicrobiaceae* seem to stabilize their metabolism by increasing the expression of Mtr and Mcr to preserve the ion motive force for energy conservation with *Methanomicrobiaceae* being more successful. However, protein abundances detected by proteome analysis do not necessarily reflect the presence and activity of functional enzyme complexes. Subunits of TE-dependent enzyme complexes might also be expressed at elevated levels to compensate the increasing number of non-functional enzymes under TE-limiting conditions.

Our study has shown how methanogens react to TE deprivation by adapting their energy metabolism and suggests that *Methanosarcina* and *Methanoculleus* use different strategies to cope with such a limitation. Proteome analysis and tracer experiments revealed that *Methanosarcina* shifted from acetoclastic to hydrogenotrophic methanogenesis while *Methanoculleus* increased the hydrogenotrophic activity to sustain energy conservation. *Methanosarcina* as the versatile and multipotent “heavy duty” methanogen (De Vrieze et al., 2012) is more fastidious with regard to TE supplementation than *Methanoculleus*, which is a sufficient substitute not only as partner for SAOB but also as more robust methanogen stabilizing reactor performance under critical conditions.

## Author contributions

BW and SK designed the study and the experiments. BW performed the experiments. BW, NJ, and DP analyzed the data. BW, NJ, DP, HH, and SK interpreted the data. BW drafted the manuscript and NJ, DP, HH, and critically revised it. All authors completed the final version of the manuscript and have approved it.

### Conflict of interest statement

The authors declare that the research was conducted in the absence of any commercial or financial relationships that could be construed as a potential conflict of interest.
