# Supplementary material for: Metabolic Adaptation of Methanogens in Anaerobic Digesters Upon Trace Element Limitation
Source: Front Microbiol. 2018 Mar 13;9:405. doi: 10.3389/fmicb.2018.00405 (PMC5859356; doi:10.3389/fmicb.2018.00405)
Supplement: Supplementary file 1 [file DataSheet1.pdf]

frontiers

Supplementary Material

Metabolic Adaptation of Methanogens in Anaerobic Digesters upon Trace Element Limitations

Babett Wintsche, Nico Jehmlich, Denny Popp, Hauke Harms, Sabine Kleinsteuber*

*** Correspondence:** sabine.kleinsteuber@ufz.de

1 Supplementary Data

The Excel file **'Data Sheet 2.xlsx'** contains information on the number of reads (spreadsheet "Sequences") and the operational taxonomic units (spreadsheet "OTUs") identified by 454 sequencing of *mcrA* amplicons.

The Excel file **'Data Sheet 3.xlsx'** contains all datasets on protein fractions assigned to the methanogenic pathways of *Methanomicrobiaceae* and *Methanosarcinaceae*. Ratio of methanogenic proteins and total proteins are listed in the spreadsheet 'Methanogenic proteins'. The following spreadsheets list the protein fractions for each methanogenic enzyme detected in batch cultures from reactor R1 and R2 at the four sampling times (week 65, 77, 80, 84). Each spreadsheet contains the original values according to the raw data ("Raw") and the values normalized by the protein ratios given in the spreadsheet 'Methanogenic proteins'.

2 Supplementary Figures

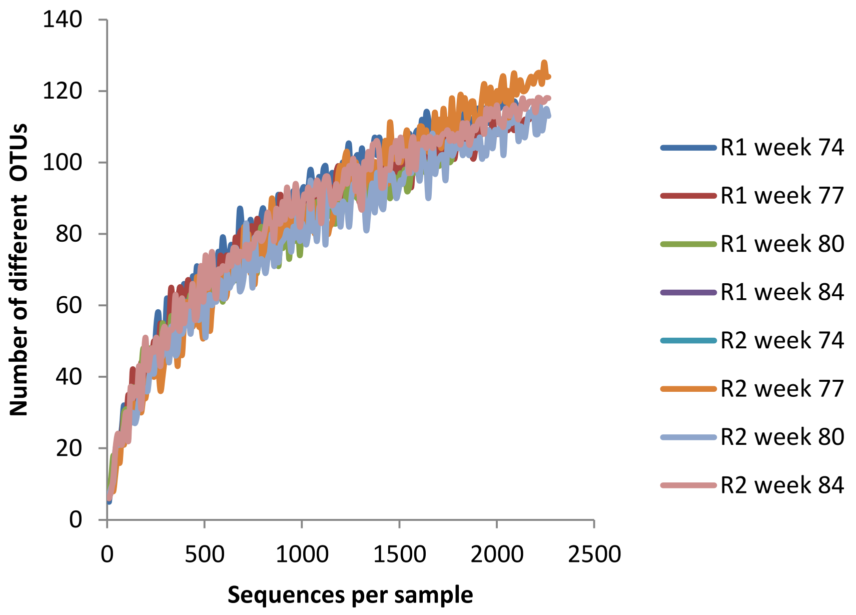

Figure S1. Rarefaction curves showing the coverage of the total richness of methanogens based on 454 reads of *mcrA* amplicons.

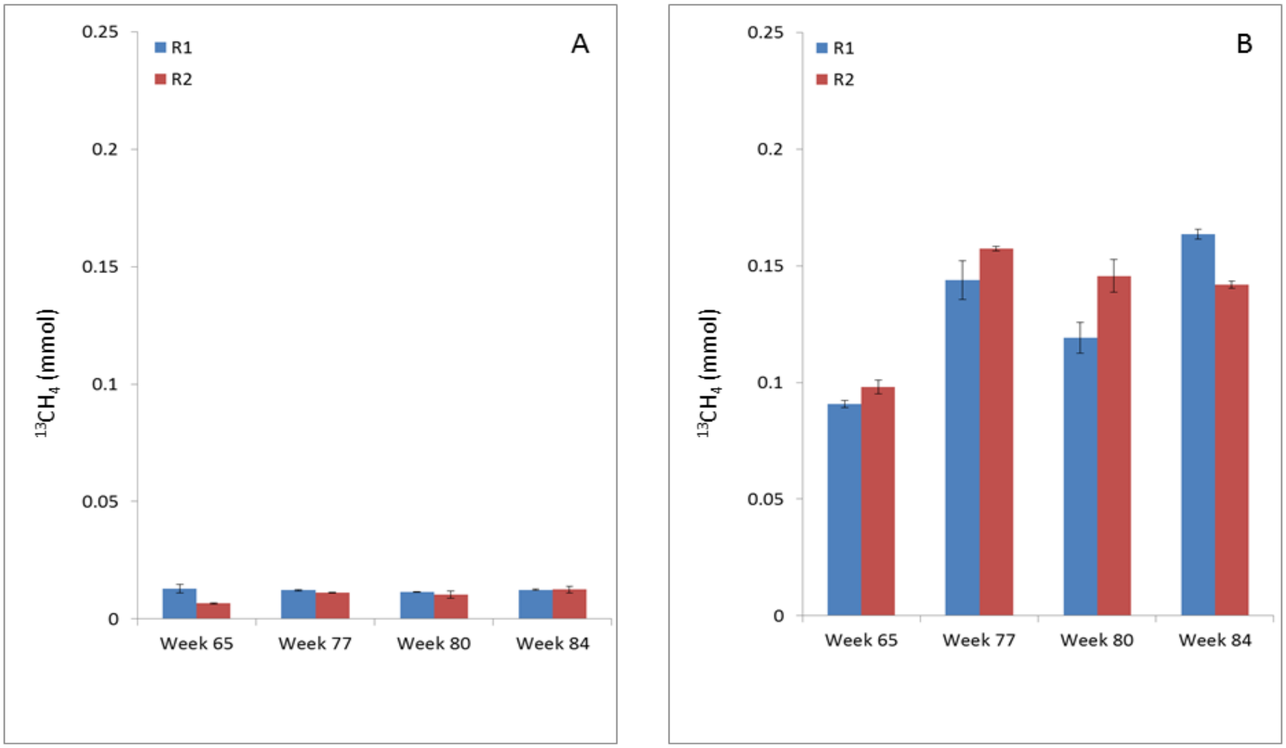

Figure S2. Formation of ^{13}C-labeled methane from 0.25 mmol ^{13}C-methyl-labeled acetate in batch cultures set up from reactor R1 (control) and reactor R2 (TE-depleted reactor) after 0 h (A) and 8 h (B). Error bars indicate standard deviation of triplicate measurements.

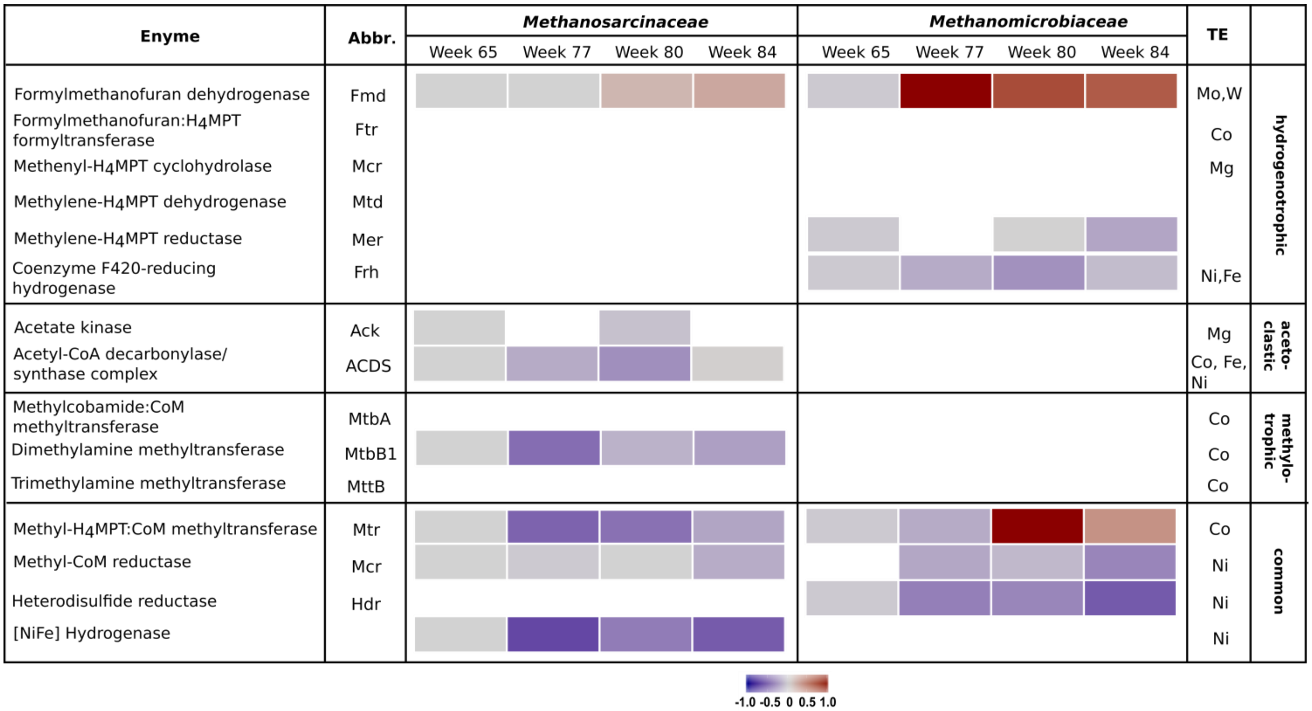

Figure S3. Heatmap of enzyme abundances of the methanogenic pathways employed by *Methanosarcinaceae* and *Methanomicrobiaceae* over the four sampling times in reactor R1. For each enzyme, the specific methanogenic pathway and the required trace elements are given. Grey bars indicate initial conditions, blue bars declining protein abundances, and red bars increasing protein abundances. Missing bars indicate enzymes that were not detected.

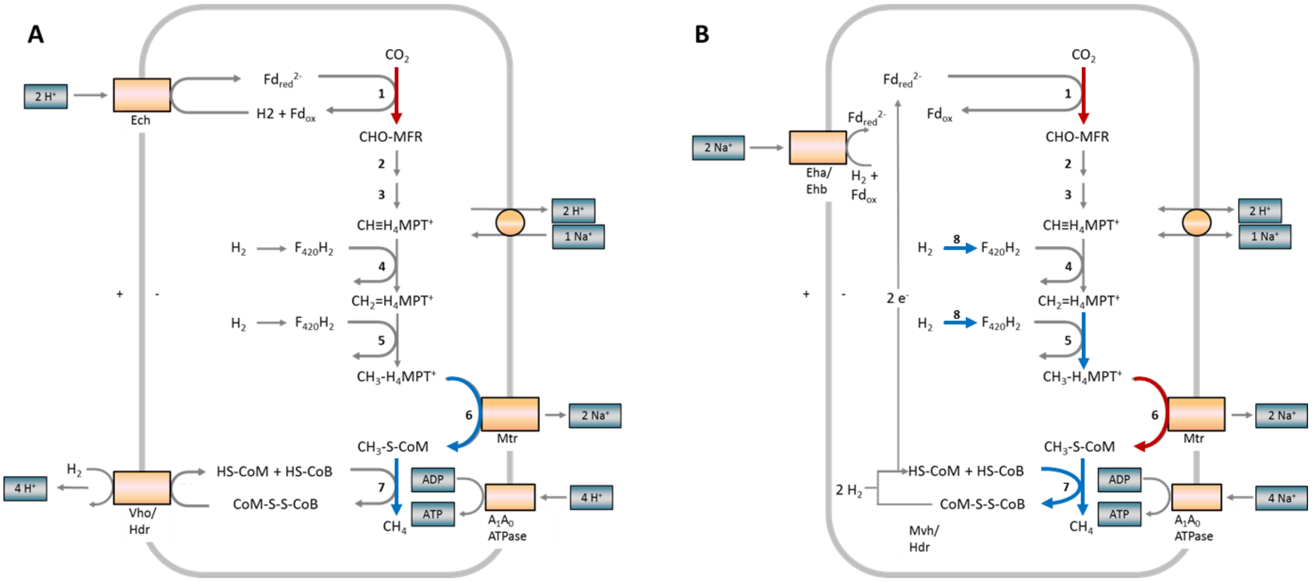

Figure S4. Scheme of hydrogenotrophic methanogenesis in *Methanosarcinaceae* (A) and *Methanomicrobiaceae* (B) and observed protein abundance shifts in samples from reactor R1 (week 84). The numbers at the reaction arrows correspond to the reaction numbers in **Table 1** (see main text). Colored arrows indicate changing protein abundances between week 65 and week 84. Red arrows indicate increasing protein abundances, blue arrows decreasing protein abundances, gray arrows show proteins not detected. Arrow thickness indicates the protein abundances. Thick = more abundant, thin = low abundant. (A) In *Methanosarcina*, the first and last steps of methanogenesis are chemiosmotically coupled and ATP generation is driven by a proton motive force. (B) In *Methanoculleus*, the first and last steps of methanogenesis are coupled by flavin-based electron bifurcation and ATP generation is driven by a sodium motive force. Fd, ferredoxin; MFR, methanofuran; H_4MPT, tetrahydromethanopterin; HS-CoM, coenzyme M; HS-CoB, coenzyme B; Ech, FeS hydrogenase; Vho/Hdr, F_{420} non-reducing hydrogenase/heterodisulfide reductase; Eha/Ehb, energy-converting hydrogenase complex; Mvh/Hdr, methyl-viologen-reducing hydrogenase
